# Cyr61-positive cancer stem-like cells enhances distal metastases of pancreatic cancer

**DOI:** 10.18632/oncotarget.12248

**Published:** 2016-09-26

**Authors:** Weidong Shi, Chenyue Zhang, Zhen Chen, Hao Chen, Luming Liu, Zhiqiang Meng

**Affiliations:** ^1^ Department of Integrative Oncology, Fudan University Shanghai Cancer Center, Shanghai 200032, China; ^2^ Department of Oncology, Shanghai Medical College, Fudan University, Shanghai 200032, China; ^3^ Collaborative Innovation Center for Cancer Medicine, Fudan University Shanghai Cancer Center, Shanghai 200032, China

**Keywords:** Cyr61, cancer stem-like cells, metastases, pancreatic cancer

## Abstract

Efficient inhibition of tumor metastasis after resection of primary tumors is critical for cancer therapy. We have recently shown that Cyr61 promotes growth of pancreatic ductal adenocarcinoma (PDAC) through PI3k/Akt signaling-enhanced nuclear exclusion of p27. Here, we report that administration of adeno-associated viral vectors carrying a short-hairpin interfering RNA (shRNA) for Cyr61 via pancreatic duct significantly decreased the distal tumor metastases after resection of primary pancreatic tumor in mice. Moreover, Cyr61 depletion in PDAC cells significantly inhibited the tumor sphere formation *in vitro*, significantly decreased the growth of the subcutaneously transplanted tumor, and significantly decreased the incidence of tumor formation after serial adoptive transplantation into NOD/SCID mice. Finally, higher Cyr61 levels were detected in the PDAC specimens from the patients with distal tumor metastasis, compared to PDAC without metastasis at diagnosis. Together, our study suggests that suppression of Cyr61 in cancer stem cell-like cells in PDAC may inhibit tumor cell metastasis after resection of the primary tumor.

## INTRODUCTION

Pancreatic ductal adenocarcinoma (PDAC) is one of the most lethal cancers with an extreme low 5-year survival rate of the patients [1, 2]. A gene called K-ras has been shown as a genetic driver of pancreatic cancer initiation and progression [3]. Moreover, combined mutation of pancreatic K-ras and p53 leads to development of PDAC-like tumor in mice, which has been widely used for studying PDAC [4, 5].

The surgical removal of the primary tumor has been proposed to have both beneficial and adverse effects upon cancer spread and growth [6]. Nevertheless, paradoxical results have been obtained from clinical data on outcome of surgical removal of the primary tumor, seemingly as a consequence of preventing the patient from dying of a locally invasive primary tumor, and thus allowing distant metastasis to grow out [6–10]. In some cases, the removal of the primary tumor may result in growth of the metastatic cancer in the distal area [6]. Wound fluids contain a number of pro-angiogenic factors and those pro-angiogenic factors in wound fluids also enter circulation and then promote neo-angiogenesis in the distal area containing metastatic cancer cells [7–10].

However, the growth of distal metastatic tumor may rely on a specific group of cancers cells, which are called cancer stem cells (CSCs). CSCs have characteristics of stem cells, and are tumorigenic, and responsible for cancer relapse and metastasis [11–14]. Treatments targeting CSCs, evident from rodent studies, are suggested to improve the therapeutic outcome on rapidly growing cancers and highly metastatic cancers [11–14]. Although cell surface markers are generally used for isolation of CSCs by flow cytometry, none of these CSC-markers have been found to be 100% specific, in which these markers are believed to enrich CSCs from a certain tumor, but are not able to purify CSCs. Therefore, most characterized “CSCs” are actually CSC-like cells [15–19]. So far, the gold standard to identify CSCs or CSC-like cells is by tumor sphere formation in a limiting dilution assay and by tumor formation in serial adoptive transplantation [20–22].

Cysteine-rich protein 61 (Cyr61) is a growth factor-inducible, immediate-early gene that regulates cell adhesion, chemostasis, growth factor-mediated DNA synthesis, foster cell survival and angiogenesis [23–25]. Cyr61 has been shown to be involved in the regulation of a number of signaling pathways, and thus its biological function is diverse. Upregulation of Cyr61 has been found in different cancers, and is associated with tumor malignancy [26–30]. For example, Cyr61 interacts with NF-κB signaling [31], or with Src signaling [32], to promote tumorigenesis in breast cancer. In gastric and colorectal carcinoma, Cyr61 binds to integrin to activate extracellular-related kinase/mitogen-activated protein kinase (ERK/MAPK) and phosphatidylinositol 3-kinase (PI3K) to increase cell growth [33, 34]. However, Cyr61 has been shown to be a tumor suppressor in lung cancer [35, 36]. These data suggest that the effects of Cyr61 on carcinogenesis may be tumor-specific. Recently, we report Cyr61 activation in the pancreatic cancer xenograft [37], in the human pancreatic cancer cell lines [38], and in the pancreatic carcinoma [39]. Moreover, Cyr61 seemed to activate PI3K signaling pathway to induced nuclear exclusion of p27 to increase PDAC proliferation [40]. However, unlikely in gastric and colorectal carcinoma [33, 34], Cyr61 does not regulate ERK/MAPK signaling in PDAC [40]. Here, we address the question whether Cyr61 may play a role in the distal metastases of PDAC after primary tumor removal, which is a critical but unstudied question.

## RESULTS

### Schematic of *in vivo* experiment and AAV vectors

**Figure 1:**
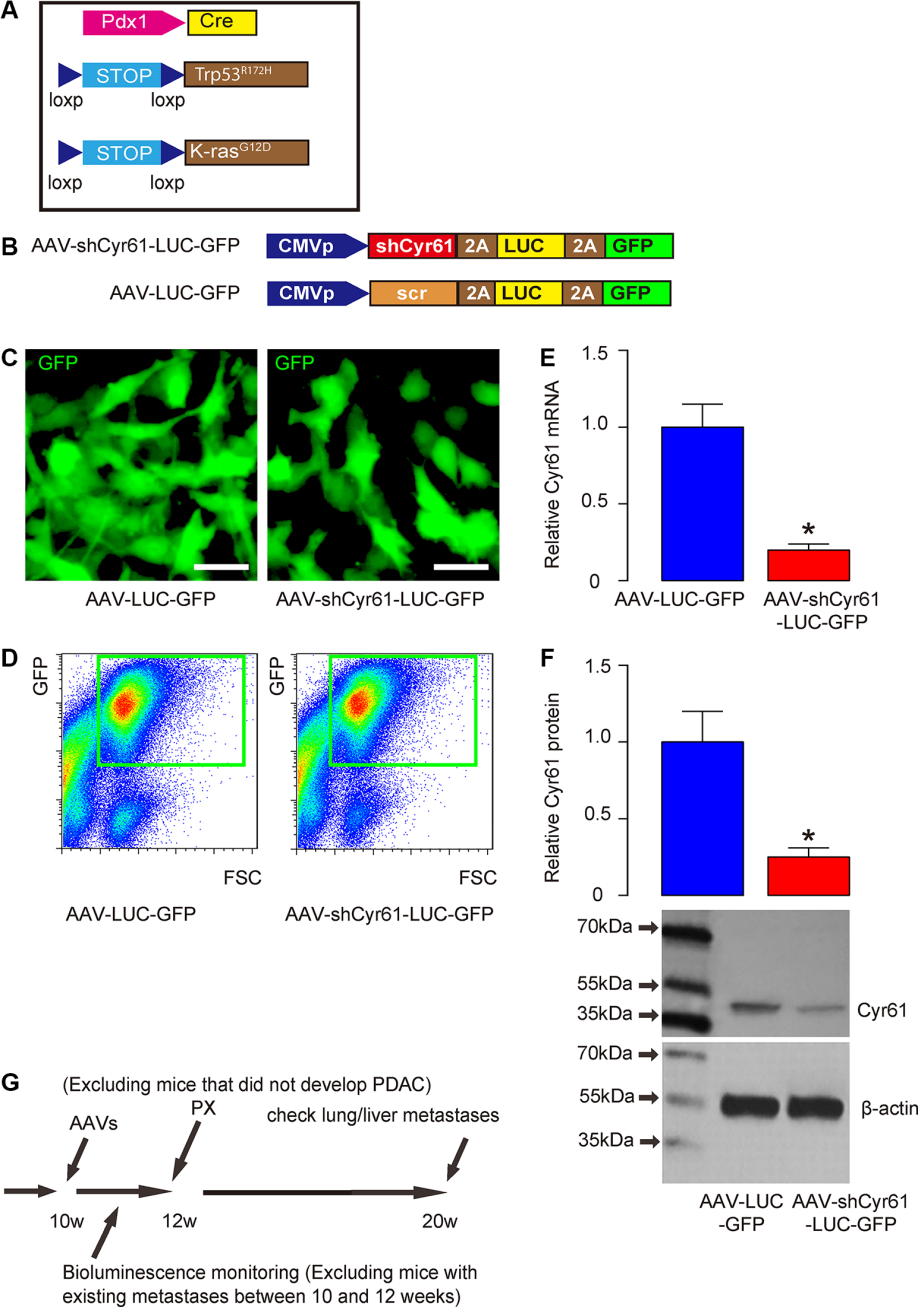
Schematic of *in vivo* experiment and AAV vectors (**A**) Schematic of triple mutant male mice (Pdx1-Cre, Conditional Loxp-STOP-Loxp (LSL)-Trp53^R172H/+^ and LSL-Kras^G12D^/+). (**B**) Schematic of AAV virus (AAV-shCyr61-LUC-GFP and AAV-LUC-GFP). (**C**) A human PDAC cell line, PANC-1, was transduced with AAVs and the cells appeared green due to expression of GFP in culture. (**D**) To obtain purified cells, the transfected cells were further subjected to flow cytometry to isolate GFP+ cells, shown by representative flow charts. (**E**–**F**) Cyr61 levels in the purified GFP+ cells by RT-qPCR for mRNA (E) and by Western blot for protein (F). (**G**) Mice received intraductal infusion of AAV at 10 weeks of age. The mice were kept for 2 weeks before pancreatectomy (PX) was performed. During the 2 weeks, bioluminescence was monitored and all mice that showed bioluminescence outside pancreas region (e.g. lung and liver) were excluded from the study. At the time of PX, mice that did not develop PDAC were also excluded from the study. Afterwards, the mice received insulin pellets and were kept for another 8 weeks, before they were lively analyzed for presence of distal metastases (at liver and lung) through bioluminescence and then sacrificed to analyze presence of distal metastases through examination of the GFP mRNA levels in liver and lung. *p < 0.05. *N* = 20. Scale bars are 10 μm.

We used a mouse model for PDAC, in which the PDAC-developing rate is more than 90% and the phenotype has been shown to very close of PDAC in humans [4]. Triple mutant male mice were thus generated (Pdx1-Cre, Conditional Loxp-STOP-Loxp (LSL)-Trp53^R172H^/+ and LSL-Kras^G12D^/+; Figure 1A). Then, adeno-associated virus (AAV) carrying short-hairpin interfering RNA for Cyr61 (shCyr61) and two reporters, luciferase and GFP (simplified as AAV-shCyr61-LUC-GF), and control AAV carrying a scrambled shRNA and the two reporters Thus, two AAVs (simplified as AAV-LUC-GFP) were prepared (Figure 1B). We used a human PDAC cell line, PANC-1, to examine the quality of these viruses. PANC-1 cells were transduced and appeared green due to expression of GFP in culture (Figure 1C). To obtain purified cells, the transfected cells were further subjected to flow cytometry to isolate GFP+ cells (Figure 1D). The purified GFP+ cells were then checked for the alteration of Cyr61 levels. We found that Cyr61 depletion by shRNA significantly reduced both the mRNA (Figure 1E) and protein level (Figure 1F) in PANC-1 cells by more than 80%. These data confirmed that quality of Cyr61-depleted AAV. Then, we performed the *in vivo* experiments. The mice received intraductal infusion of the AAVs, and then were kept for 2 weeks before pancreatectomy (PX) was performed. During the 2 weeks, bioluminescence was monitored and all mice that showed bioluminescence outside pancreas region (e.g. lung and liver) were excluded from the study. At the time of PX, mice that did not develop PDAC were also excluded from the study. Afterwards, the mice received s.c. implantation of Insulin pellets to maintain glucose metabolism. The mice were then kept for another 8 weeks, before they were lively analyzed for presence of distal metastases (at liver and lung) through bioluminescence and then sacrificed to analyze presence of distal metastases through examination of the GFP mRNA levels in liver and lung (Figure 1G).

### Quality control of intraducal infusion of AAV in mouse pancreas and examination of PDAC formation

**Figure 2:**
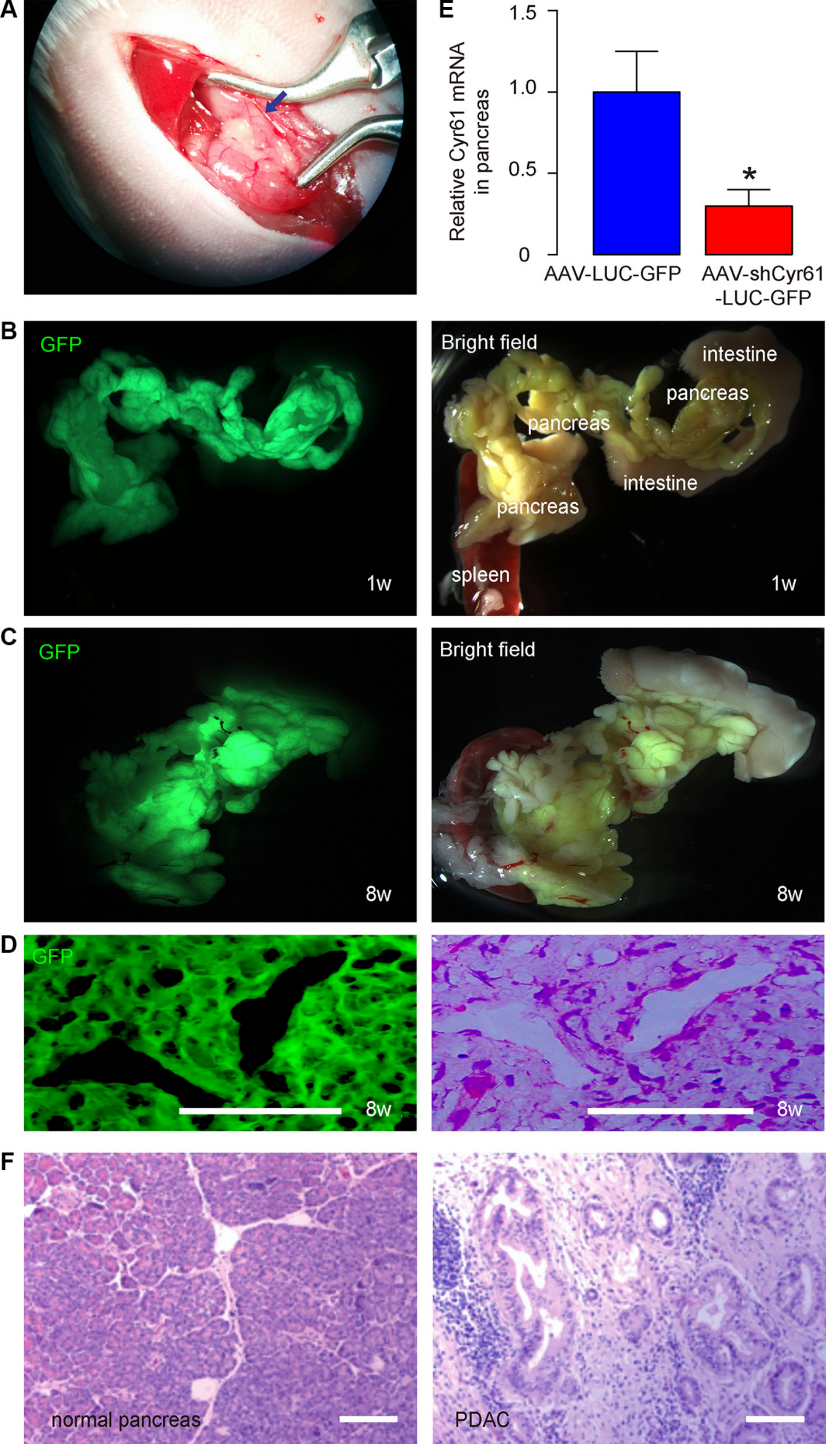
Quality control of intraducal infusion of AAV in mouse pancreas and examination of PDAC formation (**A**) Illustration of surgical performance of intraductal infusion of the pancreas. The blue arrow points to the blunt end of the catheter inside the duct. (**B**–**C**) Representative gross images of mouse pancreas 2 days (B) and 8 weeks (C) after intraductal infusion. GFP and bright fields are shown. Anatomic markers are labeled. (**D**) Immunostaining for GFP on tumor tissue in pancreas section 8 weeks after AAV infusion (left panel) and H&E staining on a consecutive section (right panel). (**E**) RT-qPCR for Cyr61 in mouse pancreas 2 days after intraductal infusion. (**F**) Presence of PDAC based on H&E staining shown by representative images. *p < 0.05. *N* = 20. Scale bars are 50 μm.

We used a recently published model for suppressing Cyr61 expression in pancreas cancer. The AAV-shCyr61-LUC-GFP and AAV-LUC-GFP were infused into mouse pancreas via pancreatic duct as described originally [41] and as in several related studies [42–44] (Figure 2A). Two days after infusion, the pancreas appeared to be green fluorescent due to the presence of GFP in the AAV (Figure 2B). Moreover, the GFP was still present in the pancreas 8 weeks after viral infusion, suggesting permanent transduction of the pancreatic cells and the tumor cells in the pancreas (Figure 2C). The mRNA was taken from the pancreas and showed that infection by AAV-shCyr61-LUC-GFP significantly reduced Cyr61 levels (Figure 2D). Two weeks after infusion, the PX was performed and the removed pancreas was examined for presence of PDAC based on histology (Figure 2E). Then, the mice were kept for another 8 weeks, before they were lively analyzed for presence of distal metastases (at liver and lung) through bioluminescence and then sacrificed to analyze presence of distal metastases through examination of the GFP mRNA levels in liver and lung.

### Cyr61 suppression reduces the chances of distal metastatic tumor formation after PX

**Figure 3:**
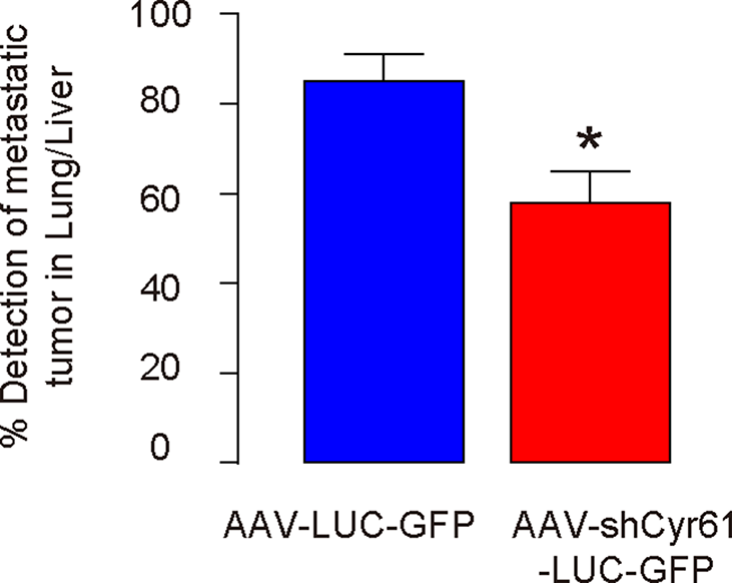
Cyr61 suppression reduces the chances of distal metastatic tumor formation after PX Rate of forming detectable metastatic tumors in either liver or lung 8 weeks after PX (removal of original PDAC) in mice that had received AAV-shCyr61-LUC-GFP, compared to the mice that had received AAV-LUC-GFP. *p < 0.05. *N* = 20.

We found that the mice that had received AAV-shCyr61-LUC-GFP had a significantly lower rate of forming detectable metastatic tumors in either liver or lung 8 weeks after PX (removal of original PDAC), compared to the mice that had received AAV-LUC-GFP (Figure 3). Thus, Cyr61 suppression reduces the distal metastatic tumor formation after PX.

### Cyr61 suppression reduces CSC-like cells *in vitro*

**Figure 4:**
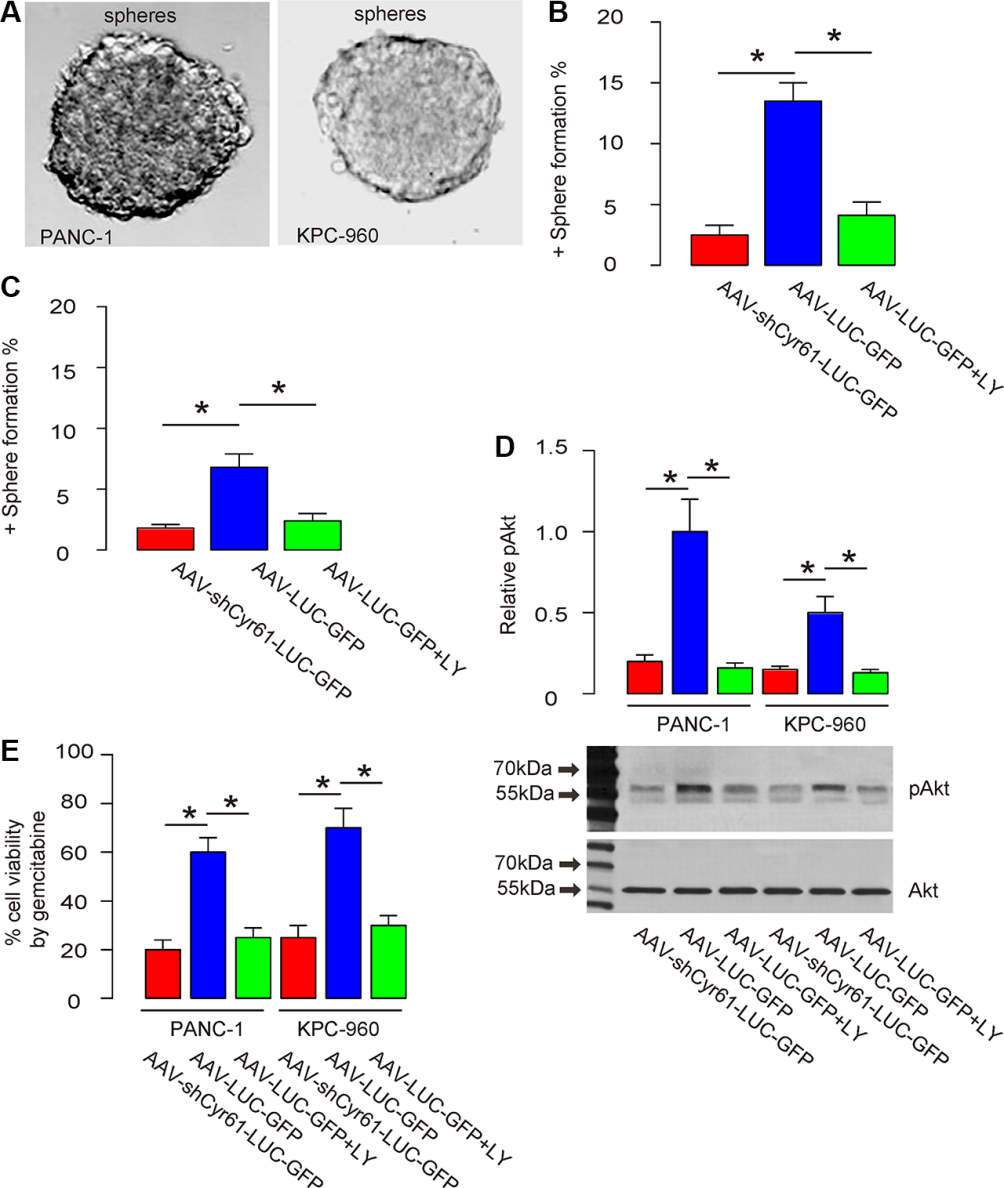
Cyr61 suppression reduces CSC-like cells *in vitro* (**A**) Representative positive or negative sphere formation. (**B**–**C**) Percentage of the wells that form tumor sphere in a limited dilution assay of PANC-1 cells (B) or KPC-960 cells (C). (**D**) Western blot for Akt phosphorylation. (**E**) Cell viability in a CCK-8 assay at presence of gemcitabine. LY: LY29400. pAkt: phosphorylated Akt. *p < 0.05. *N* = 5.

Next, we examined the underlying mechanisms. Since CSCs play a critical role in the formation of metastatic tumor, we checked the effects of Cyr61 on CSCs in pancreatic cancer. We used 2 most commonly used human PDAC cell line, PANC-1 and KPC-960. The PANC-1 or KPC-960 cells that were transduced with either AAV-shCyr61-LUC-GFP or AAV-LUC-GFP were subjected to a tumor sphere limiting dilution assay. We found that AAV-shCyr61-LUC-GFP-transduced PANC-1 cells grew into sphere-like structures in a significantly lower ratio than AAV-LUC-GFP-transduced PANC-1 cells, by representative images (Figure 4A), and by quantification (Figure 4B). Similarly, AAV-shCyr61-LUC-GFP-transduced KPC-960 cells grew into sphere-like structures in a significantly lower ratio than AAV-LUC-GFP-transduced KPC-960 cells, by representative images (Figure 4A), and by quantification (Figure 4C). These seemed to result from the abolishment of Akt activation as shown in our previous study [40], since administration of a specific inhibitor for Akt phosphorylation, LY294002 (Figure 4D), into the culture of either cells transduced with AAV-LUC-GFP, mimicked the effects of Cyr61 depletion on sphere formation in the sphere forming media (Figure 4B–4C), and on the cell viability at presence of a chemotherapeutic drug, gemcitabine (Figure 4E).

### Cyr61 suppression reduces tumor growth *in vivo*

**Figure 5:**
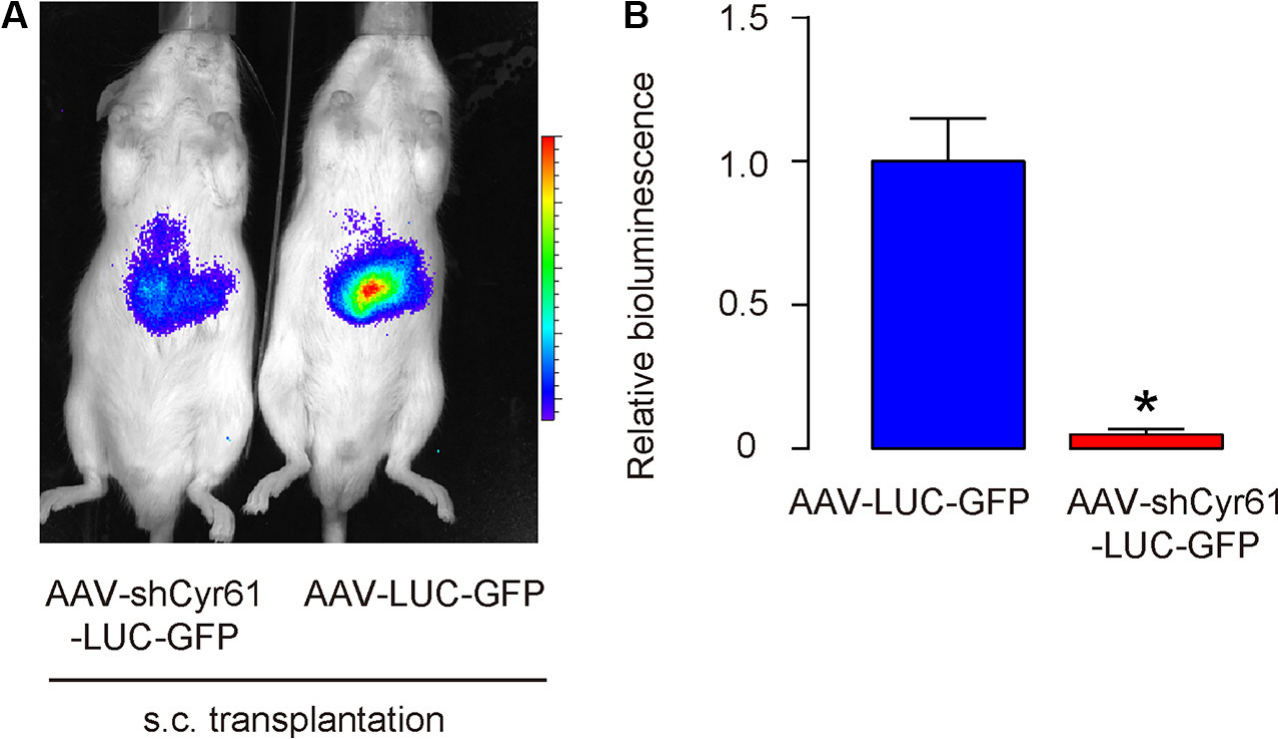
Cyr61 suppression reduces tumor growth *in vivo* (**A**–**B**) Tumor cells (10^6^) were s.c. implanted into NOD/SCID mice and the mass of the formed tumor was analyzed 6 weeks after transplantation, based on bioluminescence examination, shown by representative images (A), and by quantification (B). *p < 0.05. *N* = 20.

Moreover, when same number of these cells (10^6^) were s.c. implanted into NOD/SCID mice, AAV-shCyr61-LUC-GFP-transduced PANC-1 cells developed much smaller tumor, compared to AAV-LUC-GFP-transduced PANC-1 cells, 6 weeks after transplantation, based on bioluminescence examination, shown by representative images (Figure 5A), and by quantification (Figure 5B). These data suggest that Cyr61 suppression reduces tumor growth *in vivo*.

### Cyr61 suppression reduces occurrence of tumor formation in serial adoptive transplantation

**Figure 6:**
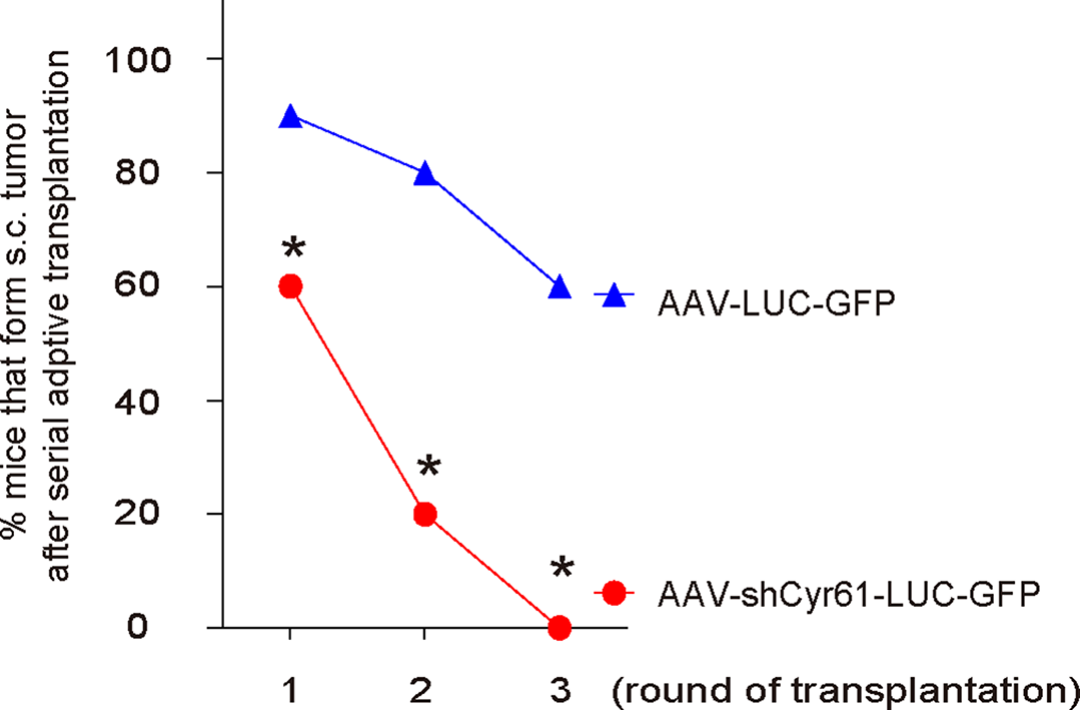
Cyr61 suppression reduces occurrence of tumor formation in serial adoptive transplantation A hundred tumor cells from s.c. tumor by either AAV-shCyr61-LUC-GFP or AAV-LUC-GFP –transduced PANC-1 cells were transplanted s.c. to new NOD/SCID mice and the formation of tumor was examined by bioluminescence after 6 weeks. The confirmed tumor was then dissected and used for the 100 tumor-cell-transplantation for the next round. Three rounds of transplantation were performed. The rate of developing tumor was analyzed, based on bioluminescence examination. *p < 0.05. *N* = 20.

Another gold standard for determining CSC-like cells is potent of tumor formation after serial adoptive transplantation. Thus, 100 tumor cells from s.c. tumor by either AAV-shCyr61-LUC-GFP or AAV-LUC-GFP -transduced PANC-1 cells were transplanted s.c. to new NOD/SCID mice and the formation of tumor was examined by bioluminescence after 6 weeks. The confirmed tumor was then dissected and used for the 100 tumor-cell-transplantation for the next round. Three rounds of transplantation were performed. We found that AAV-shCyr61-LUC-GFP-transduced PANC-1 cells had significantly rate of developing tumor, compared to AAV-LUC-GFP-transduced PANC-1 cells, at all 3 rounds of transplantation, based on bioluminescence examination (Figure 6). These data further support that Cyr61 suppression reduces CSC-like cells in PDAC.

### Higher Cyr61 levels are detected in PDAC specimens with distal metastasis

**Figure 7:**
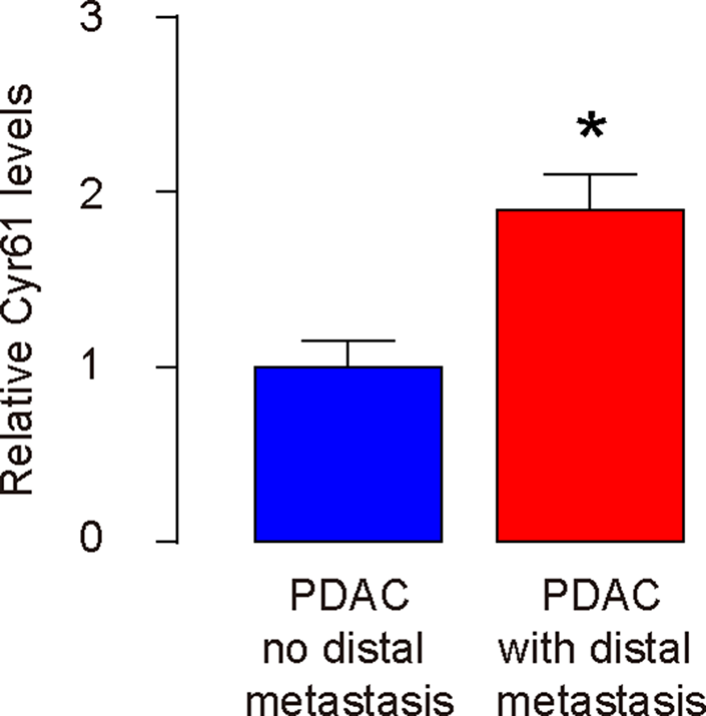
Higher Cyr61 levels are detected in PDAC specimens with distal metastasis Resected PDAC specimens (all Stage III) from 35 subjects were analyzed for Cyr61 levels by RT-qPCR and the samples were grouped based presence of distal metastasis (*n* = 19) or not (*n* = 16) at the time of diagnosis. *p < 0.05.

Finally, we examined the clinical evidence for Cyr61 as a CSC-associated factor. Resected PDAC specimens (all Stage III) from 35 subjects were analyzed for Cyr61 levels and the samples were grouped based presence of distal metastasis (*n* = 19) or not *(n* = 16) at the time of diagnosis. Higher Cyr61 levels were detected in the PDAC specimens with distal metastasis, compared to PDAC without metastasis at diagnosis (Figure 7).

## DISCUSSION

Great efforts have been made to study and characterize CSCs in pancreatic cancer. Recently, Sharma et al. reported aberrant reactivation of PI3K/Akt/mTOR and Sonic Hedgehog (Shh) signaling pathways in pancreatic CSCs. Moreover, combined inhibition of PI3K/Akt/mTOR and Shh pathways showed promising effects on the treatment of pancreatic cancer [45]. In studies from another group, Song et al. showed that significant higher levels of microRNA-21 and lower levels of FoxO1 in PDAC specimens. FoxO1 was then found to be regulated by microRNA-21 to control cancer cell proliferation, and FoxO1-low, microRNA-21-high cells had CSC-like properties [42, 46, 47]. Similarly, we have previously shown Cyr61 activation in the pancreatic cancer xenograft [37], in the human pancreatic cancer cell lines [38], and in the pancreatic carcinoma [39]. Moreover, Cyr61 seemed to activate PI3K signaling pathway to induced nuclear exclusion of p27 to increase PDAC proliferation [40]. Here, we were inspired by the studies from Song et al. [42, 46, 47], and we addressed the question whether Cyr61 may play a role in the distal metastases of PDAC after primary tumor removal, and whether Cyr61 may be a marker of CSC-like cells in PDAC.

A combined mutation of K-ras and p53 has been shown to develop PDAC-like tumor efficiently in mice [4, 5]. In the current study, we used this model to evaluate the role of Cyr61 suppression in pancreatic cancer cell metastases after primary tumor removal, which was fulfilled by PX. Since the presence of distal metastases before pancreatic Cyr61 suppression may affect the interpretation of our results, we examined the bioluminescence in mouse lung and liver between intraductal infusion of shCyr61 viruses and PX, taking advantage of the expression of luciferase in tumor cells after intraductal infusion. Intraductal infusion into the pancreas has been shown to be a powerful technology to induce gene expression or depletion specifically in pancreas, which were shown by previous studies and by us here based on fluorescence from GFP [41–44].

For determination of presence of cancer metastases, we examined lung and liver, since both are the most common metastatic sites for pancreatic cancer. We used two assays for examining the metastases. First, the metastatic cancer cells could be visualized in the regions of lung and liver in luciferin assay, since they were transduced with AAV carrying luciferase and GFP during intraductal infusion. Second, since the cancer cells also expressed GFP by intraductal infusion, the relative minor presence of them could be detected by RT-qPCR on liver and lung samples. Combination of two assays allow us to relatively objectively determine the presence of distal metastases of the pancreatic cancer.

Although cell surface markers have been widely used to isolate and purify CSCs, the gold standards for determining CSC properties are tumor sphere formation and serial adoptive transplantation. Here, we used both assays to find out that Cyr61 suppression significantly reduced the CSC-like cells in pancreatic cancer cells. These results may result either from the elimination of CSCs by removing Cyr61 in these cells, or from silencing of CSCs if Cyr61 is necessary for CSCs to maintain active CSC properties. These possibilities may be examined in the future study.

Together, based on our previous studies and data shown here, we provide evidence that Cyr61 seems to be expressed for CSC-like cells in PDAC, and Cyr61 activates PI3k signaling pathway to enhance cell growth, increase their invasiveness and resistance to chemotherapy. Suppression of Cyr61 may decrease the CSC-like feature of the PDAC cells, and thus reduce the occurrence of distal metastases after removal of primary tumor. These findings have implications for clinical application.

## MATERIALS AND METHODS

### Protocol approval

All the experimental methods in the current study have been approved by the research committee at Fudan University Shanghai Cancer Center. All the experiments have been carried out in accordance with the guidelines from the research committee at Fudan University Shanghai Cancer Center. All animal experiments were approved by the Institutional Animal Care and Use Committee at Fudan University Shanghai Cancer Center (Animal Welfare Assurance). Surgeries were performed in accordance with the Principles of Laboratory Care, supervised by a qualified veterinarian.

### Specimens from patients

A total of 35 resected PDAC specimens (all stage III; 19 with distal metastasis and 16 without) were collected for this study. All specimens had been histologically and clinically diagnosed at Fudan University Shanghai Cancer Center from 2008 to 2015. For the use of these clinical materials for research purposes, prior patient's consents and approval from the Institutional Research Ethics Committee were obtained.

### Mouse manipulation

Pdx1-Cre, Conditional Loxp-STOP-Loxp (LSL)-Trp53^R172H^/+ and LSL-Kras^G12D^/+ were interbred strains, and obtained from Jackson Labs (Bar Harbor, ME, USA) [4]. Triple mutant animals were obtained by efficient crossing. Only male mice were used for the experiments, starting at 10 weeks of age. The role of genomic instability in tumor progression remains a C57BL/6 background. Ten week-old male NOD/SCID mice (SLAC Laboratory Animal Co. Ltd, Shanghai, China) were used for subcutaneous (s.c.) transplantation of tumor cells and serial adoptive transfer. At 10 weeks of age, triple mutant mice received intraductal infusion of adeno-associated virus (AAV) carrying short-hairpin interfering RNA for Cyr61 (shCyr61) and two reporters, luciferase and GFP, or control AAV carrying a scrambled shRNA and the two reporters. AAV-serotype 6 viruses (titration of 10^12^ genome copy particles/ml and in a volume of 120 μl) were delivered via the catheter at a rate of 6 μl/min, as has been described [41]. The mice were kept for 2 weeks before pancreatectomy (PX) was performed. During the 2 weeks, bioluminescence was monitored and all mice that showed bioluminescence outside pancreas region (e.g. lung and liver) were excluded from the study. At the time of PX, mice that did not develop PDAC were also excluded from the study. Afterwards, the mice received s.c. implantation of 2 mouse Insulin pellets (LINβIT, LinShin Canada, Inc., Toronto, Canada) at the back side of the neck, according to manufacturer's instruction, to maintain glucose metabolism. The mice were kept for another 8 weeks, before they were sacrificed and analyzed for presence of distal metastases (at liver and lung) through bioluminescence and through examination of the GFP mRNA levels in liver and lung. For s.c. transplantation of pancreas cancer cells into NOD/SCID mice, 10^6^ cancer cells were implanted s.c. and the tumor formation was examined after 2 weeks by bioluminescence. For serial adoptive transplantation of cancer cells, 100 cells were isolated from formed tumor and re-transplanted s.c. into NOD/SCID mice. GFP sorting of the tumor cells were performed in every round of transplantation. The tumor formation was examined after 6 weeks by bioluminescence.

### Tumor monitoring by bioluminescence

Formation of tumor at s.c. sites and in lung and liver was monitored by luciferin assay, based on luciferase activity of tumor cells. Bioluminescence was measured with the IVIS imaging system (Xenogen Corp., Alameda, CA, USA). All of the images were taken 10 minutes after intraperitoneal injection of luciferin (Sigma-Aldrich) of 150 mg/kg body weight, as a 60-second acquisition and 10 of binning. During image acquisition, mice were sedated continuously via inhalation of 3% isoflurane. Image analysis and bioluminescent quantification was performed using Living Image software (Xenogen Corp.).

### Cell line culture

PANC-1 and KPC-960 cells were both purchased from American Type Culture Collection (ATCC, Rockville, MD, USA). Both cell lines were cultured in Dulbecco's modified Eagle's medium (DMEM) supplemented with 20% fetal bovine serum (Invitrogen, Carlsbad, CA, USA), 4.5 g/l glucose, and penicillin/streptomycin mixture (Sigma-Aldrich, St. Louis, MO, USA) in a humidified chamber with 5% CO_2_ at 37°C. LY294002 (Sigma-Aldrich) was applied to the culture at 20 μmol/l at a frequency of 12 hours till the end of experiment. Gemcitabine (Sigma-Aldrich) was applied to the culture at 100 nmol/l.

### Plasmid transfection

The construct for shCyr61 was purchased from Santa Cruz Biotechnology (Dallas, Texas, USA). The shCyr61 or null and a 2A sequence were sub-cloned into a pAAV-CMV-Luciferase-2A-GFP plasmid backbone (Clontech, Mountain View, CA, USA), with a packaging plasmid carrying the serotype 6 rep and cap genes and a helper plasmid carrying the adenovirus helper functions (Applied Viromics, LLC. Fremont, CA, USA) to generate AAVs in this study using Lipofectamine 2000 reagent (Invitrogen). The small 2A peptide sequences, when cloned between genes, allow for efficient, stoichiometric production of discrete protein products within a single vector through a novel “cleavage” event within the 2A peptide sequence. Sequencing was performed to confirm the correct orientation of these new plasmids. The viruses were purified using CsCl density centrifugation and then titration was determined by a quantitative densitometric dot-blot assay. For cell transduction *in vitro*, the cells were incubated with AAV at a MOI of 100 for 12 hours. Stable transduced cells expressing shCyr61 or control null were selected by flow cytometry based on GFP. Transduced cells were monitored *in vivo* by their expression of luciferase.

### Tumor sphere culture with a limiting dilution assay

Cancer cells were washed, acutely dissociated in oxygenated artificial cerebrospinal fluid and subject to enzymatic dissociation. To investigate the percentage of single cells capable of regenerating new spheres, cancer cells were re-suspended in tumor sphere media (TSM) consisting of a serum-free DMEM, human recombinant Epidermal growth factor (20 ng/ml; Sigma-Aldrich), bFGF (20 ng/ml; Sigma-Aldrich), leukemia inhibitory factor (10 ng/ml; Sigma-Aldrich) and N-acetylcysteine (60 μg/ml; Sigma-Aldrich), and then plated at a dilution of 1–2 cell per well in a 96-well plate. The number of the wells per 100 wells that form tumor spheres was quantified.

### Cell counting kit-8 (CCK-8) assay

The CCK-8 detection kit (Sigma-Aldrich) was used to measure cell viability according to the manufacturer's instructions. Briefly, cells were seeded in a 96-well microplate at a density of 5000/ml. After 24h, cells were treated with resveratrol. Subsequently, CCK-8 solution (20 ml/well) was added and the plate was incubated at 37^°^C for 2 h. The viable cells were counted by absorbance measurements with a monochromator microplate reader at a wavelength of 450 nm. The optical density value was reported as the percentage of cell viability in relation to the control group (set as 100%).

### Histology

After the mouse pancreas was removed by PX, the pancreas was fixed in 4% formalin for 6 hours, followed by cryo-protection in 30% sucrose overnight before freezing in a longitudinal orientation (from tail to head of the pancreas) and sectioned at 6 μm. HE staining was then performed to examine presence of PDAC. In each animals, at least 10 slides that were 50 μM in distance were checked to exclude the mice that did not carry PDAC.

### Quantitative real-time PCR (RT-qPCR)

Total RNA was extracted from mouse tissue or cultured cells with RNeasy kit (Qiagen, Hilden, Germany) for cDNA synthesis. RT-qPCR was performed in duplicates with QuantiTect SYBR Green PCR Kit (Qiagen). All primers were purchased from Qiagen. Data were collected and analyzed using ^2−ΔΔCt^ method for quantification of the relative mRNA expression levels. Values of genes were first normalized against β-actin, and then compared to the experimental controls.

### Western blot

Protein was extracted from the cultured cells with RIPA lysis buffer (Sigma-Aldrich) on ice. The supernatants were collected after centrifugation at 12000 × g at 4°C for 20 min. Protein concentration was determined using a BCA protein assay kit (Bio-rad, China), and the proteins were separated on SDS-polyacrylamide gels, and then transferred to a PVDF membrane. The membrane blots were first probed with a primary antibody. After incubation with horseradish peroxidase-conjugated second antibody, autoradiograms were prepared using the enhanced chemiluminescent system to visualize the protein antigen. The signals were recorded using X-ray film. Primary antibodies were rabbit anti-Cyr61, anti-phosphorylated Akt (pAkt), anti-Akt and anti-β-actin (Cell Signaling, San Jose, CA, USA). Secondary antibody is HRP-conjugated anti-rabbit (Jackson ImmunoResearch Labs, West Grove, PA, USA). β-actin was used as a protein loading control. The protein levels were first normalized to internal controls, and then normalized to the experimental controls.

### Statistical analysis

All of the statistical analyses were performed using the GraphPad Prism 6 (GraphPad Software, San Diego, CA, USA). Statistical analysis of group differences was carried out using a one-way analysis of variance (ANOVA) test followed by followed by Tukey multiple comparison post-hoc analysis. All values represent the mean ± standard deviation (SD). A value of *p* < 0.05 was considered statistically significant after Bonferroni correction.
